# Using Zebrafish to Model Autism Spectrum Disorder: A Comparison of ASD Risk Genes Between Zebrafish and Their Mammalian Counterparts

**DOI:** 10.3389/fnmol.2020.575575

**Published:** 2020-11-11

**Authors:** Victoria Rea, Terence J. Van Raay

**Affiliations:** Dept of Molecular and Cellular Biology, University of Guelph, Guelph, ON, Canada

**Keywords:** ASD, autism, genes, behavior, zebrafish, human, mice

## Abstract

Autism spectrum disorders (ASDs) are a highly variable and complex set of neurological disorders that alter neurodevelopment and cognitive function, which usually presents with social and learning impairments accompanied with other comorbid symptoms like hypersensitivity or hyposensitivity, or repetitive behaviors. Autism can be caused by genetic and/or environmental factors and unraveling the etiology of ASD has proven challenging, especially given that different genetic mutations can cause both similar and different phenotypes that all fall within the autism spectrum. Furthermore, the list of ASD risk genes is ever increasing making it difficult to synthesize a common theme. The use of rodent models to enhance ASD research is invaluable and is beginning to unravel the underlying molecular mechanisms of this disease. Recently, zebrafish have been recognized as a useful model of neurodevelopmental disorders with regards to genetics, pharmacology and behavior and one of the main foundations supporting autism research (SFARI) recently identified 12 ASD risk genes with validated zebrafish mutant models. Here, we describe what is known about those 12 ASD risk genes in human, mice and zebrafish to better facilitate this research. We also describe several non-genetic models including pharmacological and gnotobiotic models that are used in zebrafish to study ASD.

## Introduction

Autism spectrum disorders (ASDs) are a group of heterogenous neurodevelopmental disorders caused by both genetic and environmental factors. ASD hallmarks include restricted interests, repetitive behavior, social and learning impairments, and sensory hyperactivity or hypoactivity (Belmonte et al., 2004; Jeste and Geschwind, 2014). Diagnosis of ASDs is defined by the standards in *Diagnostic and Statistical Manual of Mental Disorders, 5^*th*^ edition* (American Psychiatric Association, 2013). The genetic complexity and pleiotropic nature of ASDs, combined with a number of potential environmental causes has made the etiology of ASD difficult to elucidate and has hampered the development of potential therapies (Bölte et al., 2019; Courchesne et al., 2019). Approximately 1–3% of all autism cases are associated with maternally derived duplications of the 15q11-q13 region; largely including Prader-Willi and Angelman syndromes (Depienne et al., 2009). Mutations in known ASD loci such as in Fragile X and Rett syndrome are present in 4–5% of ASD cases (Reddy, 2005; Richards et al., 2015). While ASDs are considered highly heritable disorders, heritability only accounts for an estimated 50% of ASDs and concordance is not 100% even in monozygotic twins (Tammimies, 2019). Furthermore, the overlap of symptoms and manifestations present in different syndromic and idiopathic forms of ASDs makes it difficult to unravel ASD etiology as well as separate potential treatments. Recent transcriptomic studies have revealed that neuronal development and maturation of the immune system are both associated with ASD and cellular and molecular pathways such as synaptic function and WNT signaling have been identified through enrichment analyses (Quesnel-Vallières et al., 2019). A recent whole-exome sequencing study of ASD found many genes affect the synapse and are expressed early in the excitatory inhibitory neuronal lineages (Satterstrom et al., 2020). The authors also note that most ASD risk genes are responsible for regulation of expression of other genes. Another recent study points to insufficient myelination caused by dysregulation of oligodendrocytes associated with ASD in both mice and humans (Phan et al., 2020). ASD are also associated with environmental factors. The maternal and infant microbiome, antibiotic treatment, and epigenetic changes have all been associated with the development and progression of ASD pathogenesis. For example, individuals with ASD are known to have gastrointestinal problems and distinct microbiome taxonomic profiles compared to neurotypical (NT) individuals (Sgritta et al., 2019). Though the current understanding of the association between the microbiome and ASD is limited, recent data shows strong correlations between gut microbiota and the etiology of ASD, with bacterially-derived metabolites from the gut linked to alterations in neurodevelopment and neural specific mRNA processing of the host (Irimia et al., 2014; Stilling et al., 2018).

Though most ASD studies to date have used rodent models (Hulbert and Jiang, 2016; Sgritta et al., 2019), the zebrafish model presents an additional and useful tool in tackling the complexity and variability of ASD. Zebrafish (*Danio rerio*) are becoming a more commonly used neurodevelopmental model, as they represent a genetically tractable vertebrate species with high physiological and genetic homology to humans (Kozol et al., 2016; Shams et al., 2018). As such, zebrafish can be a powerful model to study ASD and have proved particularly useful in forward genetic screens as well as visualizing neural development (Kalueff et al., 2014; Stewart et al., 2014a; Gerlai, 2017, 2019). It is fairly easy to manipulate the zebrafish genome with tools like CRISPR and such experiments are aided further through the high fecundity of zebrafish, which allows for large samples sizes typically unavailable in mammalian models. Furthermore, external fertilization, rapid development and optical transparency provide early access to developmental stages that aren’t as quickly and easily available in the mouse model. For example, between 2- and 3-days post-fertilization (dpf) in zebrafish, primary neurons are replaced through secondary neurogenesis and major parts of the brain begin to establish and differentiate. This stage is equivalent to embryonic stage [E] 12.5 to 13.5 in mice where expression of proneural genes and other transcription factors are comparable between the two model organisms (Wullimann, 2009). Recent reviews highlight zebrafish development and techniques available to study neurodevelopmental disorders (Kozol et al., 2016; Sakai et al., 2018). Furthermore, with the recent advantages of making embryos germ-free, zebrafish offer a semi high-throughput and cost-effective animal model that is highly suitable for studying the environmental and idiopathic aspects of ASD. In this review, we focus the initial 12 ASD risk genes identified by the Simons Foundation for Autism Research Initiative (SFARI) to facilitate ASD research using zebrafish and describe what we know about these genes in humans, mice and zebrafish.

## Zebrafish as a Model Organism

**FIGURE 1:**
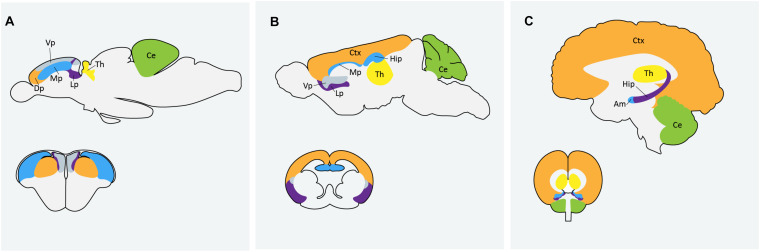
Comparison of homologous regions of the **(A)** zebrafish **(B)** mouse and **(C)** human brains. Am: amygdala; Ce: cerebellum; Ctx: cortex; Dp: dorsal pallium; Hip: hippocampus; Lp: lateral pallium; Mp: medial pallium; Th: thalmus; Vp: ventral pallium. Zebrafish image in **(A)** adapted from Mueller et al. (2011) and Mueller (2012).

Zebrafish are becoming an increasingly popular model organism to study neurodevelopment and neurological disorders. The zebrafish genome shares over 70% homology with human genes and neurodevelopmental processes are conserved between zebrafish and humans (Howe et al., 2013). A recent analysis by Meshalkina et al. (2018) found approximately 62% of the 858 human ASD risk genes listed in the SFARI database as having zebrafish orthologs. Zebrafish and mammalian brains are similar in macro-organization and cellular morphology (Kozol et al., 2016). Zebrafish possess all major neural cell types including neurons, astrocytes, oligodendrocytes and microglia and regions that are often associated with ASD, like the human cortex and amygdala, are homologous to the zebrafish dorsal and medial pallium respectively (Figure 1). There are some differences in the way the zebrafish brain is set up during development, particularly during neurulation. Rather than direct folding of the neural plate to form the neural tube, as is the process in mammals, zebrafish first form a solid neural keel which then inflates into the neural tube during secondary neurulation (Schmidt et al., 2013). However, the cells present during this process are topologically arranged in a similar manor to that of other vertebrates and ultimately result in the formation of a highly similar structure. Because of the whole-genome duplication in teleost fish, duplication of some genes in the zebrafish genome persist, and subsequent human genes may have two zebrafish orthologs; the obvious disadvantage being possible functional redundancy, but possible advantage being sub-functionalization of phenotypes (Glasauer and Neuhauss, 2014). It can also be an asset in studying genes with mammalian embryonic lethality, where mutation in only one of the zebrafish paralogs results in a simpler phenotype with embryos remaining viable (Glasauer and Neuhauss, 2014). Alternatively, this challenge can be easily overcome by creating loss-of-function mutations in both paralogs using CRISPR technologies (Jao et al., 2013).

Zebrafish also have well-characterized social behaviors and widely accepted social tests that have yet to be applied in an ASD context, though changes in locomotor activity are often seen in zebrafish genetic models of ASD (Ng et al., 2013; Kim et al., 2017). Shoaling behavior and various social preference tests have been suggested as possible tests to model ASD-like behaviors in zebrafish though caution should be taken with using behavior to validate gene function, particularly in studying such a pleiotropic group of disorders. A list of widely accepted behavioral tests for zebrafish research is outlined in Box 1 and the use of zebrafish as a behavioral neuroscience model has been covered in several excellent reviews (Kalueff et al., 2014; Stewart et al., 2014a; Gerlai, 2017, 2019).

**Behavioral tests for zebrafish research**

Below are various widely accepted behavioral tests for zebrafish. It is important to note that behavioral tests can be affected or confounded by multiple factors including strain (for example AB wild-type fish generally show more of a preference for social novelty than wild caught Indian strains), familiarity with the environment, temperature and pH of the water, light/dark cycle, etc. (Gerlai, 2019).

**Visually mediated social preference test:** This test is designed to measure social preference and social novelty of adult zebrafish using a five-chamber tank, infrared light source and standard video camera. Test fish in the center compartment are surrounded by 2 control compartments, familiar fish and unfamiliar fish. Time spent near conspecifics, speed, and distance traveled are all measured to evaluate social behavior in fish, similar to social interaction tests in rodents (Norton et al., 2019).

**3-Chamber social choice test:** Similar to the visually mediated social preference test, the three chamber social choice test uses a center chamber, and a chamber on either side; one containing fish that were acclimated with the controls and the other chamber is originally left empty during acclimation, and naive fish are placed in the chamber during phase two (Ariyasiri et al., 2019). This test is also performed on adult fish.

**Shoaling behavior:** Shoaling is a social behavior necessary for zebrafish survival and requires the recognition of conspecifics. The impairment of coordination of conspecific activity can be likened to the impaired social interaction of individuals with ASD (Neri, 2012). Zebrafish form shoals, or clustered groups, in order to forage, avoid predators or find mates (Engeszer et al., 2007). Shoaling behavior starts to emerge at 6 days post-fertilization and throughout the first 21 days, zebrafish establish strong social preference for conspecifics through visual stimuli (Meshalkina et al., 2018). Shoaling behavior can be measured using two video cameras to allow 3D-video recording. Fish are acclimated to tank apparatus and various measurements are recorded in real time. These include nearest neighbor distance, farthest neighbor distance, average inter-individual distance, and time spent both in and outside the shoal. It is helpful to measure the same behaviors in a control group in parallel (Meshalkina et al., 2018).

**Thigmotaxis:** Thigmotaxis, or “wall-hugging” was first used as a behavioral measurement for rats but is now a validated measurement of anxiety in zebrafish and zebrafish larvae, which can be measured as early as 5 days post-fertilization (Schnörr et al., 2012). Increased anxiety, represented by increased thigmotaxis, is measured by the amount of time spent in the center zone of the well or tank with respect to the perimeter and the number of entries into the center zone. Less entries into the center zone or less time spent in the center zone, and therefore increased thigmotaxis, represents higher anxiety levels (Phelps et al., 2017).

## Zebrafish Models of ASD

Zebrafish are a useful animal model for both genetic and non-genetic instances of ASD. ASDs affect neural circuitry and social behaviors that are evolutionarily conserved. For example, it is relatively easy to visualize migrating axons in real time (Rieger et al., 2011) thus, zebrafish models can be used to both strengthen and even enhance the research conducted in rodents and humans. Zebrafish possess cognitive responses similar to those observed in humans and rodents including phenotypes related to social interactions, social preference, repetitive behaviors and cognitive inflexibility (Box 1) (Stewart et al., 2014b). Several monogenic zebrafish models of ASDs have been created and are available for distribution from the Zebrafish International Resource Center (ZIRC) and unlike rodent models, zebrafish knockout models of ASD risk genes can be investigated using only one gene in an ortholog pair (Glasauer and Neuhauss, 2014). Furthermore, zebrafish are highly useful in pharmacological studies as compounds can be administered through simple water immersion (Goldsmith, 2004). The zebrafish hypothalamic-pituitary-interrenal (HPI) axis is homologous to the human hypothalamic-pituitary-adrenal (HPA) axis, which is strongly associated with autism (Alsop and Vijayan, 2009). Zebrafish also harbor similar neuroendocrine systems to humans and release cortisol as a stress hormone; contrary to rodents that release corticosterone (Canavello et al., 2011). Overall, zebrafish represent a highly useful tool in dissecting the underlying nature of ASDs.

## Genetic Models

The Simons Foundation for Autism Research Initiative has created a curated list of zebrafish lines with mutations in 12 ASD risk genes https://www.sfari.org/resource/zebrafish-models/. While not an exhaustive list there is general consensus as to their involvement in ASD. Loss of function has been independently validated in all 12 of these genes and five lines are currently available for distribution (*ARID1B, CHD8, FMR1, MECP2*, and *PTEN*). Importantly, all of these loss-of-function lines are not validated by phenotypic behavior, but by direct measurement of target mRNA or protein levels. The remaining 7 (*CNTNAP2, DYRK1A, GRIN2B, NRXN1, SCN2A, SHANK3*, and *SYNGAP1*) have loss-of-function models that have been independently validated but are not yet available through the ZIRC. Nonetheless, zebrafish harboring mutations for these genes exist and here we briefly describe the current status and recent advances of these 12 genes and their relationship with the zebrafish models for Autism research. SFARI has an established gene scoring system based on the strength of evidence associating each gene with risk of autism. ASD risk genes can be classified into one ‘syndromic’ category or one of three ‘idiopathic’ categories: category 1 (high confidence), 2 (strong candidate), or 3 (suggestive evidence). SFARI gene scores are dynamic and each gene score associated with the below genes correspond to their score at time of publication. A large proportion of the mutations that inform ASD risk gene score are found through whole-exome sequencing. The most recent and largest exome sequencing study in ASD to date implicates 102 genes in risk for ASD (Satterstrom et al., 2020).

## Knock-Out Versus Knock-Down

Common methods to create loss of function models in zebrafish include genomic knockout via mutagenesis and morpholino knockdown. Morpholino oligomers can be injected into early stage zebrafish embryos and transiently knockdown the function of corresponding target genes by binding complementary target mRNAs and blocking translation, similar to siRNAs. It has been shown that genetic compensation often occurs in mutants, which can obscure potential phenotypic effects, whereas morpholino knockdown can reveal the effects of loss of protein function without inducing compensation (Rossi et al., 2015). The disadvantage of using morpholinos lies in the difficulty of discerning phenotypes caused by specific binding to the intended target RNA from the non-specific binding to unintended targets; though there are methods to help control this (Eisen and Smith, 2008; Stainier et al., 2017). Subsequently, best practice is typically to validate morpholino phenotypes in mutants (Stainier et al., 2017). Rather than targeting RNA like morpholino oligomers, the CRISPR/Cas9 system affects genomic DNA which allows for analysis of molecular affects at the single embryo level as well as to generate stable mutant lines (Harrison et al., 2014). While the CRISPR/Cas9 system is the most recently developed tool and arguably the most frequently used, other mutagenesis approaches like TALEN (Transcription activator-like effector nucleases) and ENU (*N*-ethyl-*N*-nitrosourea) mutagenesis affect DNA and are often used to create null alleles in zebrafish studies. TALENs have a larger target potential in that every 1–3 base pairs in the zebrafish genome can be targeted (Varshney and Burgess, 2014), but are restricted to simple mutations, and are more likely to result in mosaicism (Wang et al., 2013; Varshney et al., 2015). ENUs are a chemically mediated mutagenesis tool that generates randomly distributed point mutations. As such, they generate unbiased mutations in a highly efficient and very high throughput manner. However, it can be very difficult to identify these mutations and clone target genes (Harrison et al., 2014; Varshney et al., 2015). While each of the aforementioned tools have their advantages and disadvantages, morpholino’s should be evaluated with caution and considered as complementary to genome editing techniques, not as a replacement for them so long as they follow the current best practices (Stainier et al., 2017).

### ARID1B


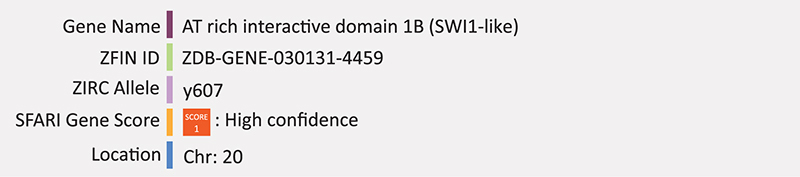


*ARID1B* encodes the AT rich interactive domain 1B protein; a subunit of the BRG1-associated factors (BAF) chromatin remodeling complex (also known as SWI/SNF complex), which regulates neurite outgrowth (Weinberg et al., 2013; Choi et al., 2015). ARID1B has also been shown to repress Wnt/β-catenin signaling through a BRG1-dependent mechanism in the nucleus at the level of β-catenin (Vasileiou et al., 2015). Variants of this gene are also responsible for Coffin-Siris syndrome (CCS), which often co-occurs with symptoms of ASD. Nord et al. (2011) previously showed that individuals with ASD have reduced *ARID1B* transcript levels. Haploinsufficiency of *ARID1B* in humans leads to ASD (Moffat et al., 2019). *ARID1B* deficient neurons have significantly reduced dendritic innervation in cortical layer I, therefore it was hypothesized that loss of ARID1B function may result in ASD through a disrupted balance of excitatory and inhibitory inputs (Ka et al., 2016; Moffat et al., 2019). A recent study in *Arid1b* knockout mice show reduced GABAergic interneuron numbers owing to increased apoptosis and decreased proliferation of progenitors (Jung et al., 2017). Two other studies report *Arid1b* heterozygous mice have hydrocephalus, consistent with some individuals with ASD (Celen et al., 2017; Shibutani et al., 2017). One of these studies also reported changes in expression levels of genes involved in axonal guidance and glutamate receptor signaling pathways (Celen et al., 2017). All three studies report behavioral phenotypes consistent with ASD-like behaviors (Celen et al., 2017; Jung et al., 2017; Shibutani et al., 2017).

The zebrafish *arid1b* loss-of-function model was created in the Harold Burgess lab through CRISPR and loss-of-function was validated through quantitative PCR (SFARI). The mutant line contains a small 13-base pair deletion in exon two, causing a frameshift mutation that results in a premature stop codon. This line, *arid1b^*y*607/+^ (AB)*, is now available through ZIRC, though to our knowledge there have been no published reports of its use. Liu et al. (2020), created a knockdown model of *arid1b* in zebrafish using morpholino oligonucleotide injection and analyzed embryonic growth as growth impairment is a major clinical feature of *ARID1B* mutations in humans. They also analyzed patients harboring pathogenic variants of *ARID1B* (Liu et al., 2020). The authors found zebrafish embryos with knockdown in *arid1b* had both significantly reduced body length and trunk defects as well as perturbed expression of osteogenic genes and chondrogenic genes. These results were consistent with the below average height found in all human patients. Furthermore, the team found perturbed Wnt/β-catenin signaling in both *arid1b* knockdown embryos and *ARID1B* knockout ATDC5 cells. These results suggest that *ARID1B* may regulate growth through the Wnt/β-catenin pathway as *ARID1B* is involved in regulating cell proliferation and differentiation (Nagl et al., 2007; Yan et al., 2008). Further, *arid1b* has previously been shown to repress Wnt/β-catenin signaling downstream of the destruction complex (Vasileiou et al., 2015). Summary: *ARID1B* mutations result in an imbalance of excitatory and inhibitory inputs linked to perturbed Wnt signaling. Zebrafish morphants have a similar short stature phenotype and perturbed Wnt signaling, but detailed molecular neurogenic analysis is lacking.

### CHD8


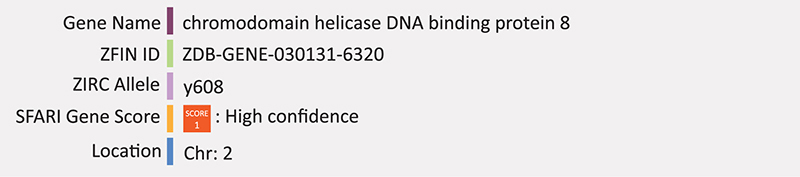


Chromatin helicase DNA-binding protein 8 (CHD8) is a transcriptional repressor that negatively regulates Wnt signaling through binding with β-catenin (Sakamoto et al., 2000; Okerlund and Cheyette, 2011; Durak et al., 2016). Truncating mutations in *CHD8* are among the high risk factors for ASD and 12 *de novo* loss-of-function mutations in *CHD8* have been reported in individuals with ASD to date (O’Roak et al., 2012, 2014; Bernier et al., 2014; Iossifov et al., 2014). Interestingly, many other ASD risk genes are known targets of *CHD8* including *ARID1B, ADNP, ASH1L, CUL3, DYRK1A, PTEN, RELN, SHANK3, SCN2A, SETD5*, and *SYNGAPP1* (Möhrle et al., 2020). Mice with *Chd8* mutations often have macrocephaly and exhibit broad gene expression changes throughout the brain. Furthermore, downregulated Wnt/β-catenin signaling has been shown in *chd8* mutant mice (Durak et al., 2016).

A *cdh8* mutant zebrafish line (*chd8*^*y*608/+^) was also created in the Harold Burgess lab through a CRISPR mediated 5-base pair deletion in the fourth exon, causing a frameshift mutation in the resulting transcript. This line was validated through QPCR and created by the same team as the *Arid1b* mutant line (SFARI). Again, to our knowledge there are no published reports of its use at the time of writing. However, two independent studies previously used morpholino injection to knockdown zebrafish *chd8* (Bernier et al., 2014; Sugathan et al., 2014). Sugathan et al. (2014) found that morpholino mediated knockdown of *chd8* results in macrocephaly (expansion of the forebrain/midbrain); consistent with CHD8 loss of function in human ASD cases (Sugathan et al., 2014). Similarly, Bernier et al. (2014) reported that disruption of zebrafish *chd8* is consistent with instances of the human phenotype including macrocephaly and reduction in post-mitotic enteric neurons leading to impairment of gastrointestinal motility, which is consistent with gastrointestinal complaints in ASD patients (Bernier et al., 2014). Summary: CHD8 is a chromatin binding protein that targets many other genes related to ASD, likely through Wnt/β-catenin resulting in macrocephaly. Zebrafish morphants have a similar phenotype but the knockout model awaits more detailed analysis.

### FMR1


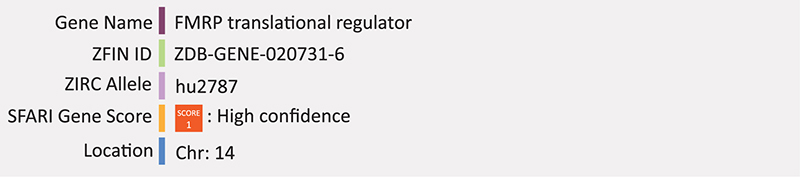


Fragile X syndrome (FXS) is a result of loss of expression of the Fragile X syndrome mental retardation 1 (*FMR1*) gene most commonly through expansion of a CGG triplicate repeat located in the 5′ untranslated region. This expanded repeat is thought to result in hypermethylation of the X chromosome in males and active X chromosome in females (Bardoni et al., 2001). The protein product, FMRP, controls the production of other neuronal proteins at synapses by binding their coding region and repressing translation (Sunamura et al., 2018). Through this mechanism, FMRP is thought to interact with pre- and post-synaptic proteins important for synaptic plasticity (Sunamura et al., 2018). While Fragile X syndrome itself is separate from autism, it is the leading monogenic cause of autism, as a significant proportion of individuals with FXS also meet the clinical diagnostic criteria for ASD (Feinstein and Reiss, 1998) and mutations in *FMR1* are present in up to 5 percent of ASD cases (Reddy, 2005). Symptoms of individuals with FXS include anxiety, intellectual disability, and hypersensitivity and males typically display more severe symptoms than females due to the X-linked nature of the genes inheritance (Kazdoba et al., 2014). Mouse models of FXS recapitulate the behavioral characteristics of FXS and both *Fmr1* knock-out mice and post-mortem brain material from FXS patients show increased density of dendritic spines (Irwin et al., 2002).

Two mutant alleles for zebrafish *fmr1* have been created via *N*-ethyl-*N*-nitrosourea (ENU) mutagenesis (hu2787 and hu2898); both introduce a point mutation, one in exon five, creating a truncated polypeptide via a premature stop codon and the other in the seventh intron creating a splice site mutation (den Broeder et al., 2009). Validated by Western blot, both alleles lack fmr1 protein entirely and produce viable progeny with no obvious phenotypes (den Broeder et al., 2009). This report contradicts previous *fmr1* morpholino reports that exhibit craniofacial development defects (Tucker et al., 2006). Two studies have since validated this knockout model and used it to analyze the social behavior of *fmr1* knockout zebrafish, reporting some phenotypes similar to that of human FXS including hyperactivity and memory impairment (Ng et al., 2013; Wu et al., 2017). However the two studies are inconsistent with respect to *fmr1* impact on anxiety where one study reports *fmr1* mutants to have anxiolytic behavior (Ng et al., 2013), while the other reports increased anxiety like behavior (Wu et al., 2017). Recently, a zebrafish FMRP knockdown model was created using a DNAzyme based method (Silverman, 2016; Medishetti et al., 2020). *Fmr1*-specific DNAzymes were electroporated into embryos between the 0 and 4 cell stage to enzymatically cleave complementary *fmr1* RNA in order to transiently knockdown FMRP levels (Medishetti et al., 2020). DNAzyme treatment successfully reduced *fmr1* mRNA and FMR1 protein levels for over 2 days. Behavior was observed using an untreated negative control group and a group treated with valproic acid as a positive control as valproic acid is another known model of neurodevelopmental injury and ASD-associated phenotypes (Dwivedi et al., 2019). FMRP knockdown embryos showed increased anxiety, irritability and cognitive impairments at 7 dpf, leading researchers to conclude that DNAzyme based knockdown of FMRP in zebrafish is a valid model of FXS (Medishetti et al., 2020). Summary: FMR1 mutations in humans leads to alterations in synaptic plasticity and dendritic spines. Knockdown of FMR1 in zebrafish displays craniofacial development defects and ASD-like behavior abnormalities. The knockout has some anxiety phenotypes but no obvious morphological phenotype. Detailed examination of molecular neurogenesis in the knockout and/or knockdown is lacking.

### MECP2


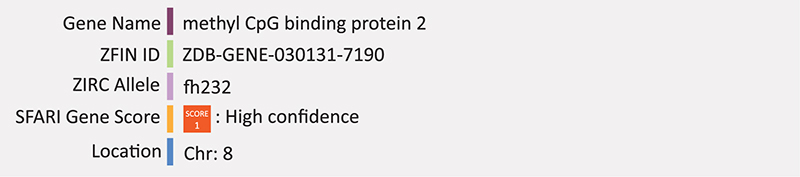


Methyl-CpG-binding protein 2 (MECP2) is a transcription factor originally described for its role in global transcriptional repression (Lewis et al., 1992). However, it was later found that *MECP2* overexpression in mice results in the majority of affected genes being activated, compared to their downregulation in *Mecp2* null mice and is now considered to have both repressor and activator activity (Chahrour et al., 2008). Since its original description, it has been revealed that MECP2 can act as both a transcriptional repressor or activator depending on its associated partners (Ragione et al., 2016), and is also likely involved in synapse maintenance, RNA-splicing and may have a wider role altogether in both neurodevelopment and adult brain function (Karaca et al., 2019). *MECP2* lies on the X chromosome and mutations in *MECP2* in hemizygous males, result in neonatal encephalopathy which is typically fatal. The same mutations in females result in a severe neurological disorder on the autism spectrum called Rett syndrome (RTT). Interestingly, duplication of *MECP2* also results in neurological disorder with similar symptoms to RTT but is typically only present in males due to the ability of the duplicated copy to be silenced during X-chromosome inactivation in females. *MECP2* is mutated in 90% of Rett-syndrome patients (Karaca et al., 2019). Rett syndrome is characterized by early cognitive regression, where individuals will typically have 6–18 months of normal development, then lose the major speech and motor skills that had been acquired (Chin and Goh, 2019). Other characteristic symptoms include gait abnormalities, microcephaly and seizures. Studies in mice highlight this genes importance in maintenance of the central nervous system rather than development, consistent with the later onset of this disease compared to other ASD associated syndromes (Pietri et al., 2013). Various *de novo* mutations in the *MECP2* gene including frameshift, missense and nonsense mutations have been found in RTT patients (Amir et al., 1999). Increased methylation of the *MECP2* promoter region is often found in ASD patients and is correlated with reduced expression of *MECP2* (Nagarajan et al., 2006). Several models of *Mecp2* dysfunction have been created in mice including knockout or null models, which have severe neuropathology similar to RTT syndrome and die at approximately 10 weeks of age. Other mutant models expressing a truncated protein, produce a slightly milder phenotype with late onset, but still have behavioral symptoms similar to that of humans and the conditional-mutant mice (Ezeonwuka and Rastegar, 2014).

The first *mecp2*-null zebrafish model was created through ENU mutagenesis [*mecp2^*fh*232/+^(AB)*] (Draper et al., 2004; Pietri et al., 2013). Loss of protein function was confirmed through western blot analysis however the mutant phenotype is mild and the authors note that this could be due in part to functional compensatory mechanisms due to the duplication of many genes in the zebrafish genome (Pietri et al., 2013). Furthermore, the zebrafish null model is viable and fertile which is inconsistent with *Mecp2*-null mouse models. Nevertheless, these fish display clear behavioral and motor abnormalities including defective thigmotaxis, increased tactile evoked response, and decrease in motor activity (Pietri et al., 2013). Recent proteomic analysis of this *mecp2*-null model has revealed 20 proteins differentially expressed in mutant compared to wild-type zebrafish at the larval stage, with the majority being under expressed in the mutant group, including many enzymes involved in glycolysis and ATP-metabolism (Cortelazzo et al., 2017). This model has also been recently used to study the role of *MECP2* in regulating the immune and inflammatory response in an RTT context. *Mecp2*-null zebrafish have increased neutrophil infiltration and downregulated expression of the proinflammatory cytokine tumor necrosis factor alpha (TNFa) (Van Der Vaart et al., 2017). Multiple groups have also tested neurodevelopment using morpholino knockdown of *mecp2* in zebrafish and report increased abnormal axonal branches of motor neurons and decreases in motor activity (Nozawa et al., 2017), inhibition of neural cell differentiation (Gao et al., 2015), and defects in peripheral innervation of sensory neurons (Leong et al., 2015). One group evaluated the knockdown of mecp2 on neural cell proliferation and migration using MO and different transgenic lines. The transgenic line Tg[*ef1a*:mAG-zGem] marked cells in S, G2 and M phase of the cell cycle; Tg[*islet1*:GFP] marked migrating motor neurons; and neural progenitor cells were identified by (Tg[*neurod*:EGFP]) (Gao et al., 2015). Overall, *neurod* and *islet1* expression were reduced in *mecp2* morphants, resulting in reduced numbers of cranial motor nerves and an increase in progenitor cells with phosphorylated histones. Taken together the researchers suggest that *mecp2* may play a role in supressing neural precursor cell differentiation (Gao et al., 2015). Summary: MECP2 is a transcriptional regulator involved in synapse maintenance and late onset phenotype. Zebrafish knockout is viable but with clear behavioral and motor deficits and uncovers a role for this gene in immune and inflammatory response. Transgenic analysis in combination with morpholino knockdown demonstrates mecp2’s role in suppressing neural precursor cell differentiation, consistent with the neuropathology seen in mice and humans.

### PTEN


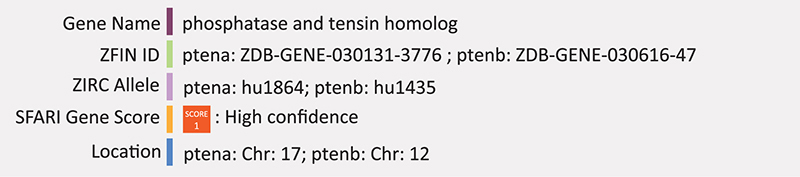


Phosphatase and tensin homolog (*PTEN*), encodes an enzyme that acts as a tumor suppressor that antagonizes phosphatidylinositol 3-phosphate kinase (PI3)/AKT signaling (Rademacher and Eickholt, 2019). Mutations in *PTEN* are often associated with syndromic cancers [together called PTEN hamartoma tumor syndromes (PHTS)], however *PTEN* is also implicated in autism as a syndromic autism gene, as mutations in *PTEN* cause Cowden syndrome and some individuals with this syndrome develop autism (Rademacher and Eickholt, 2019). Germline mutations in *PTEN* are found in up to 20% of individuals with ASD and macrocephaly (Busch et al., 2019). These mutations are typically deleterious missense or frameshift mutations, though a *de novo* loss-of-function variant has also been identified by the Autism Sequencing Consortium (De Rubeis et al., 2014). Homozygous deletions of various exons in the mouse *Pten* gene are embryonically lethal and heterozygous deletions result in widespread tumorigenesis characteristic of PHTS (Hulbert and Jiang, 2016). Conditional knockouts of *Pten* result in a phenotype that more closely resembles the human ASD-related condition even though humans with this condition have mutations in the germline (Hulbert and Jiang, 2016). For example, central nervous system deletion of *Pten* in mice shows *Pten* plays a role in controlling cell size and number pointing to a potential explanation of the etiology of associated macrocephaly. Additionally, the abnormal morphology was associated with abnormal activation of the PI3/AKT pathway suggesting these two phenotypic features may be linked (Kwon et al., 2006). Knockout of *Pten* in the neurons of mice often results in increased size and abundance of axonal projections, number of dendritic spines and number of presynaptic vesicles, as reviewed in Hulbert and Jiang (2016).

Zebrafish have two PTEN orthologs, *ptena* and *ptenb*. Mutations in each of these genes, both harboring single point mutations that are known to disrupt the enzymatic activity of human *PTEN*, have been generated (hu1864 and hu1435 respectively). These mutant lines were initially created to assess the activity of *pten* phosphatases during zebrafish embryonic development looking at the rescue capacity of *pten* but because of the genes’ association with autism, they are included in the SFARI accepted zebrafish autism models list (Stumpf and Den Hertog, 2016). The embryonic lethality of *pten* demonstrates the usefulness of the zebrafish model. In mice, *Pten* knockout is embryonically lethal at approximately day E8.5 (Di Cristofano et al., 1998) and because mice develop *in utero*, it is very difficult to study the impact of this gene during embryonic stages. Double knockout of zebrafish pten (*ptena^–/–^ptenb^–/–^*) were crossed to the transgenic line Tg(*kdrl*:eGFP), which marks the vasculature. These embryos display hyperplasia and dysplasia leading to death by approximately 5 days post-fertilization (dpf) (Choorapoikayil et al., 2013). Both *ptena* and *ptenb* can individually rescue the morphological phenotypes seen in the double mutant zebrafish (Stumpf and Den Hertog, 2016). With respect to neurodevelopment, ptena and ptenb are expressed in the cranial ganglia and brain nuclei but there has been no systematic analysis of neural phenotypes aside from altered head morphology (Croushore et al., 2005). There is evidence of its role in axon mylenation as knockdown of ptena with morpholinos activated the PI3K/Akt/mTOR pathway which resulted in an increase in axon mylenaytion (Mathews and Appel, 2016). Summary: Ptena/b is involved in controlling cell size and mutations in this gene result in enlarged axonal size and abundance, accounting for the macrocephaly phenotype. In zebrafish, knockout of ptena/b results in altered head morphology consistent with the mammalian phenotype, but as yet very little molecular neurobiology has been performed on this mutant.

The remaining seven putative loss-of-function models identified by SFARI include *CNTNAP2, DYRK1A, GRIN2B, NRXN1, SCN2A, SHANK3*, and *SYNGAP1*. Here we briefly describe what is currently known regarding their contributions to ASD research.

### CNTNAP2


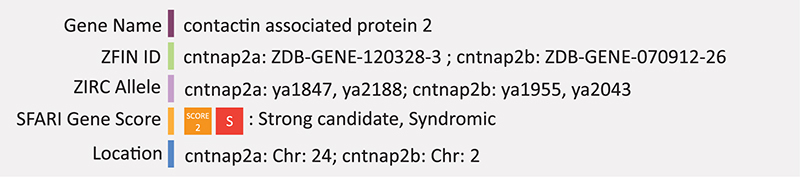


Contactin-associated protein-like 2 (CNTNAP2, also known as CASPR2) is a neuronal transmembrane protein of the neurexin family of cell adhesion proteins that function in synapse formation and regulation (Saint-Martin et al., 2018). CNTNAP2 was initially associated with ASD through its syndromic association with ASD and epilepsy (Strauss et al., 2006). *CNTNAP2* is highly expressed in humans during midgestation (Kang et al., 2011) and has been implicated in several neurological disorders including Tourette syndrome, obsessive-compulsive disorder and ASD (Tammimies, 2019). Autism-related variants in *CNTNAP2* have been shown to impair axonal growth of cortical neurons (Canali et al., 2018) and reduced excitatory and inhibitory synaptic inputs on neurons of the prefrontal cortex (Lazaro et al., 2019). *Cntnap2* mutant mice spend less time engaged in social interaction, are hyperactive, and display repetitive behaviors (measured by marble burying, digging or nest building) and excessive grooming (Möhrle et al., 2020). Alterations to the *CNTNAP2* gene associated with autism include intragenic deletions, copy number variations (CNVs), and heterozygous missense variants located throughout the gene. Mutations in the 5′ promoter region have also been identified (Chiocchetti et al., 2015; Canali and Goutebroze, 2018).

One of the largest genes in the human genome, encoding a protein with 1,331 amino acid residues, *CNTNAP2* could be considered a likely target for mutations. Zebrafish have two *CNTNAP2* orthologs, *cntnap2a* and *cntnap2b*. The primary transcript of *cntnap2a* encodes a protein of 1311 amino acids, with five known splice variants and respective proteins ranging in length from 190 to 1316 amino acids. *Cntnap2b* has two known splice variants, a primary transcript encoding a protein of 1315 amino acids, and an alternative transcript encoding a protein of 566 amino acids. Zebrafish *cntnap2a/b* proteins have 71 and 65% identity to human the CNTNAP2 protein and share the same functional domains. Hoffman et al. (2016) created a zebrafish model of *cntnap2* by inducing loss-of-function mutations in each paralog. This loss-of-function model was used in combination with behavioral profiling to identify phenotypic suppressors relevant to autism. Double mutant zebrafish show nighttime hyperactivity and increased sensitivity to drug induced seizures. Since excitatory inhibitory signaling imbalance is a major feature of ASD, the authors also compared wild-type and *cntnap2a/b* mutants using two transgenic lines that mark GABAergic and glutamatergic neurons (Tg[dlx6a-1.4kbdlx5a/dlx6a:GFP] and Tg[vglut:DsRed] respectively). Double mutant *cntnap2a/b* zebrafish had significantly fewer GABAergic cells in the forebrain and cerebellum at 4 dpf and many GABAergic cells in the ventral telencephalon failed to migrate dorsally. Interestingly, it was also found that the *cntnap2a/b* mutant behavioral phenotype was supressed by treatment with phytoestrogen biochanin A from 4 to 7 days post-fertilization suggesting the potential of estrogens as potential treatment for the disruption of neural circuits in these mutants (Hoffman et al., 2016). Summary: CNTNAP2 is a cell adhesion protein that functions in synapse formation, regulation and axonal growth. In zebrafish, mutant lines in combination with transgenic reporters identified reduced numbers of GABAergic neurons and a failure of these cells to migrate from ventral to dorsal telencephalon.

### DYRK1A


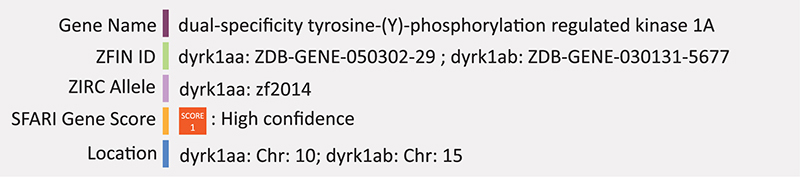


DYRK1A encodes the dual-specific tyrosine-(Y)-phosphorylation-regulated kinase 1A. DYRK1A function is dosage dependent (Duchon and Herault, 2016). Its overexpression is linked to down syndrome, but many truncating variants have been found in ASD patients and autistic behavior is included as a phenotype of DYRK1A haploinsufficiency syndrome (Dang et al., 2018). Clinical characteristics of DYRK1A associated ASD include speech and motor impairments, microcephaly, feeding difficulty and vision impairments (Earl et al., 2017). Truncating variants of DYKR1A in ASD are thought to affect the catalytic domain and result in loss of kinase activity and heterozygous mouse models have been developed to include a frameshift mutation to eliminate the kinase activity site of murine Dyrk1a (Raveau et al., 2018). These mice show fewer aggressive behaviors, less time engaging in reciprocal social behaviors, atypical vocal behaviors and experience febrile seizures like in that of human ASD-associated seizures and speech delay (Raveau et al., 2018; Möhrle et al., 2020).

Knockout of dyrk1aa in zebrafish using TALEN mediated genome editing shows social impairments relevant to autism including decreased social cohesion and anxiolytic behavior. This stable knockout line (dyrk1aa^*krb*1^) harbors a nonsense mutation leading to a truncated protein missing most of its kinase domain (Kim et al., 2017). Dyrk1aa knockout also results in microcephaly and downregulation of hypothalamic stress response markers, c-fos and crh (Kim et al., 2017). Furthermore, overexpression of either zebrafish or human DYRK1A by mRNA injection impairs development of primordial germ cells during embryogenesis, suggesting evolutionary and functional conservation in human and zebrafish (Liu et al., 2017). Zebrafish also possess another paralog dyrk1ab, however to our knowledge there have been no reports on its use as a model. Summary: DYKR1A is a tightly regulated dual kinase and loss of the catalytic domain is associated with speech and motor impairments, microcephaly feeding and vision impairments. Zebrafish mutants also have microcephaly and downregulation of the hypothalamic stress response. Detailed molecular neurobiology analysis is lacking in both mammalian and zebrafish.

### GRIN2B


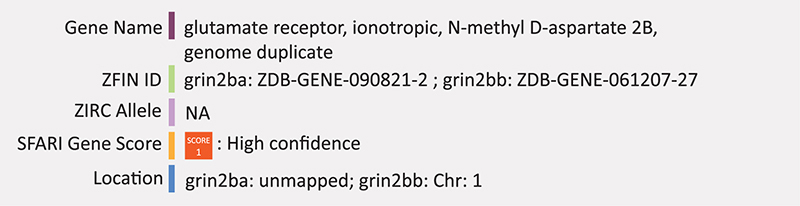


Glutamate receptor, Ionotropic, *N*-methyl D-aspartate 2b (*GRIN2B*) is one of seven of a family of genes that code for subunits of the *N*-methyl-D-aspartate receptors (NMDARs), which are ionotropic glutamate receptors found in neural cells (Myers et al., 2019). Mutations in *GRIN2B* occur in ASD and other neuropsychiatric disorders like schizophrenia and obsessive-compulsive disorder (Ohtsuki et al., 2001; Arnold et al., 2004; O’Roak et al., 2011). Expression of *GRIN2B* is highest during midgestation in the human cerebral cortex, a time of heightened neurogenesis and synaptogenesis, and then decreases with increasing fetal age suggesting an essential role in early brain development (Bagasrawala et al., 2017). Missense and nonsense mutations have been identified throughout all four domains of the human GRIN2B protein, which include the amino-terminal domain, agonist-binding domain, transmembrane domain and carboxyl-terminal domain (Myers et al., 2019). Mutations in *GRIN2B* correlate with developmental delay, language, memory and motor deficits, and sometimes seizures (Hu et al., 2016).

Mice with a homozygous *Grin2b* deletion have impaired suckling response and long-term depression leading to death at early postnatal stages (Myers et al., 2019). Heterozygous mice survive and show deficits in nest building, behavioral inflexibility (measured by spontaneous alterations in a T-maze), reduced exploratory activity and impaired spatial memory performance (Möhrle et al., 2020). Zebrafish have two GRIN2B homologs, *grin2ba* and *grin2bb*. *Grin2bb* is located on chromosome 1, however it only has 41% identity across 16% of the human encoded protein and has a more delayed expression than humans, starting at 96 h post-fertilization, well after zebrafish primary neurogenesis (Cox et al., 2005). While several point mutations and a transgenic insertion exist for this gene in zebrafish, there are no known publications on the function of this gene (knockout or knockdown) in zebrafish. *Grin2ba* is currently unmapped, however its enriched expression in hair cells eliminates its likely candidacy as an ASD-related gene (Sheets, 2017). Summary: GRIN2B is a subunit of the NMDA receptors and likely involved during the earliest stages of neurogenesis. There are no published reports of knockdown or knockout of this gene in zebrafish.

### NRXN1


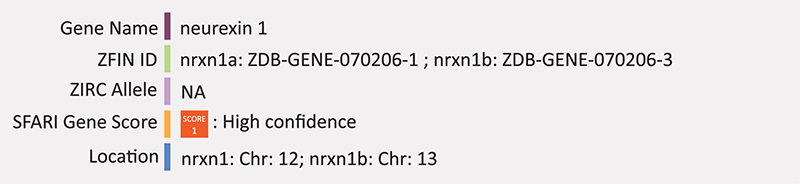


Neurexins are cell adhesion molecules of the vertebrate nervous system and are involved in synaptic cell adhesion and synaptic function. As a cell surface receptor, NRXN1 forms calcium-dependent neurexin/neuroligin complexes at synapses of the central nervous system. Hundreds of different isoforms can be produced via alternate promoters, splice sites and exons of the *NRXN1* gene, however some isoforms are predominant over others. A recent single cell analysis modeling autism in iPS neural stem cells revealed that bi-allelic deletion of *NRXN1-alpha* results in neural stem cells that tend toward radial glia-like cells rather than neurons, with a higher preference toward astroglia (Lam et al., 2019). These cells also had impaired maturation of excitatory neurons through depressed function of calcium signaling. Heterozygous microdeletions are the most common ASD-related mutations found in *NRXN1*, however diagnoses remains challenging due to variable expressivity and incomplete penetrance (Al Shehhi et al., 2019).

As ASD-related mutations in *NRXN1* are often heterozygous, most studies using mice have also used models that are *Nrxn1* heterozygous (*Nrxn1*α*^±^*). These mice exhibit sex-dependent deficits in responsiveness, memory and habituation (Al Shehhi et al., 2019). A recent study also showed hypermetabolism of the thalamic, mesolimbic and striatal regions and hypometabolism of the cortex and amygdala after ketamine administration suggesting that ketamine administration partially restores the dysconnectivity present in *Nrxn1*α*^±^* mice (Hughes et al., 2020).

Zebrafish have two NRXN1 orthologs. *Nrxn1a*, located on chromosome 12, has at least 9 alternate transcripts, 8 of which are protein coding. Phenotypes associated with this gene in zebrafish include decreases in blood circulation, angiogenesis, and caudal vain size as well as malformed caudal vein and decreased thigmotaxis. *Nrxn1b* is located on chromosome 13 and has at least two transcripts, both protein coding, but no associated phenotypes. While mutations exist (ZIRC), there have been no reports for *nrxn1* mutations related to ASD in zebrafish but morpholino knockdown of *nrxn1a* (*b-nxrn1a*) has been used to assess the formation of the vascular system during development (Rissone et al., 2012). Erythroid populations in the vasculature network of the trunk and tail were also assed in a double transgenic line Tg(gata1:dsRed)^*sd*2^;(kdrl:EGFP)^*s*843^ and β*-nrxn1a* morphants display abnormal vessles and bloodflow. Whole mount *in situ* hybridization results from this study found β-*nrxn1a* expression in diencephalon, hindbrain, and spinal cord neurons. These embryos also displayed severe impairment of locomotor activity evaluated by tail touch response and body balance maintenance (Rissone et al., 2012). Summary: NRXN1 is a synaptic adhesion protein that functions in the maturation of excitatory neurons. In zebrafish, one homolog, Nrxn1a, has impaired locomotor activity but no other ASD associated phenotypes despite significant expression in the CNS. There currently is no phenotypic information on the nxrn1b homolog.

### SCN2A


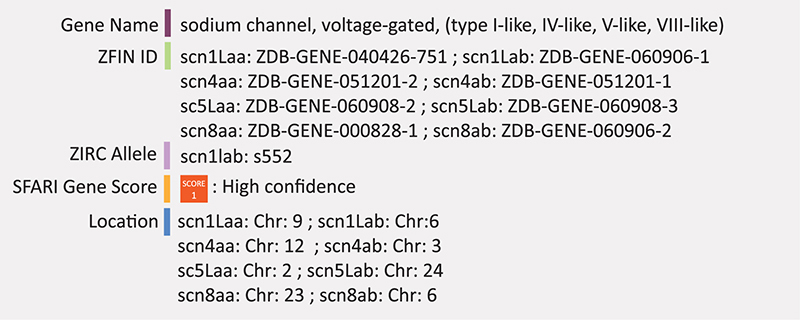


SCN2A (Sodium channel, voltage-gated, type II alpha subunit), encodes the major alpha subunit of a voltage-gated sodium channel expressed in the axon initial segment and is involved in action potential initiation and propagation. Rare variants in *SCN2A* were identified through exon screening of a group of voltage-gated sodium channel genes in autistic patients (Weiss et al., 2003). Since then, at least eight *de novo* protein truncating variants and 12 *de novo* missense variants have been described (Sanders et al., 2012; De Rubeis et al., 2014; Iossifov et al., 2014). Loss-of-function variants have been identified in individuals with ASD where loss of function of the *SCN2A* gene diminishes channel function, though the neuropathology underlying *SCN2A* associated ASD remains largely unknown (Sanders et al., 2012; Jiang et al., 2013; Tavassoli et al., 2014).

Heterozygous loss-of-function models in mice (*Scn2a*^±^) exhibit impaired action potential initiation and reduced dendritic excitability (Spratt et al., 2019). Heterozygous mice have also been shown to have impaired spatial memory (Middleton et al., 2018).

Zebrafish possess four sets of duplicated (a and b respectively) genes in the scna family: *scn1Laa/b, scn4aa/b, scn5Laa/b*, and *scn8aa/b* (Novak et al., 2006). *Scn1Laa* and *scn1Lab* are evolutionarily related to human SCN1A, SCN2A, SCN3A, and SCN9A and are expressed exclusively in the nervous system beginning at 24 hpf: *scn1Laa* in the sensory neurons of the peripheral nervous system and *snc1Lab* in the ventral regions of the hindbrain and spinal cord (Novak et al., 2006). A homozygous mutation in *scn1lab* was found through a chemical mutagenesis screen using an optokinetic response assay and originally termed *didy^*s*552^* (Schoonheim et al., 2010). The *didy*^*s*552^ loss-of-function mutation phenocopied the *scn1lab* MO loss-of-function (Schoonheim et al., 2010). Homozygous recessive loss-of-function mutation in *scn1lab* by ENU mutagenesis was later characterized in zebrafish at the molecular and behavioral level for the purpose of screening potential drugs for Dravet syndrome-related seizures that is associated with the human SCN1A gene (Baraban et al., 2013). These embryos display hyperactivity, convulsive behaviors and spontaneous abnormal electrograph activity. These embryos also exhibited reduced expression of *scn1lab*, suggesting a lack of compensation by other *scn* genes. *Scn1lab* expression was found primarily in the central nervous system, particularly in the forebrain, or telencephalon (Baraban et al., 2013). Summary: SCN1A is a voltage gated sodium ion channel involved in action potential initiation and propagation. The potential zebrafish ortholog scn1lab displays hyperactivity, convulsive behaviors and abnormal neuronal firing, consistent with known effects in humans and mice.

### SHANK3


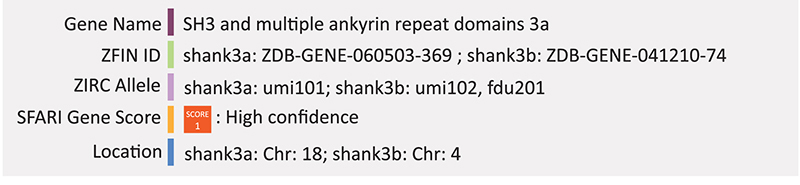


*SHANK3* has a well-established link to autism. Mutations in *SHANK3* are present in 1–25% of individuals with ASD. Both *de novo* and inherited mutations range from point mutations to whole gene deletions. The latter causes Phelan-McDermid syndrome and results in developmental and speech delays as well as severe intellectual disability accompanied by ASD (Soorya et al., 2013). Many different mutations in the *SHANK3* gene have been discovered. For example, one study screening *SHANK3* in two large cohorts of individuals with ASD (133 patients from the U.S and 88 from Italy), found different pathogenic mutations including whole gene (106 kb) deletion, two frameshift mutations leading to a pre-mature stop codon, a missense mutation, a splicing mutation and a single nucleotide polymorphism that was also detected in controls, but found in ASD cases at a much higher frequency (Boccuto et al., 2013). The C-terminus of the SHANK3 protein is required for proper targeting of SHANK3 to dendritic spines and truncating mutations in *SHANK3* negatively alter dendritic spine development and morphology (Durand et al., 2012).

Studies in mice have shown that *Shank3* is essential for proper synaptic function and mutations in this gene lead to impaired motor coordination, repetitive behavior and altered social interactions (Uchino and Waga, 2015; Amal et al., 2018; Wang et al., 2019). At least five mutant *Shank3* lines altering different regions of the protein have been created in mice (Jiang and Ehlers, 2013). Generally, *Shank3−/−* mice display autistic-like behaviors with synaptic disruption at the cortico-striatum level while isoform-specific knock-outs have varying affects (Boccuto et al., 2013).

Recently, James et al. (2019) created a *shank3* knockout model in zebrafish to test the common correlation of autism and gastrointestinal problems. CRISPR/Cas9 was used to insert a frameshift mutation in the c-terminus of the zebrafish *shank3a* and *shank3b* paralogs. Not only did these fish display a behavioral phenotype consistent with mice and humans, they also had decreased intestinal motility and peristaltic contraction rate as well as decreased number of serotonin positive enteroendocrine cells compared to wild-type (WT) and *shank3* mRNA rescue controls (James et al., 2019). Other groups have shown that loss of function mutations in zebrafish *shank3b* result in reduced locomotor activity and reduced levels of synaptic proteins (Liu et al., 2016; Liu C.X. et al., 2018). Liu C.X. et al. (2018) used CRISPR/Cas9 to create a frameshift mutation in the second exon of *shank3b* resulting in a truncated protein with all functional domains disrupted. Using the HuC-RFP transgenic line to mark differentiating neurons, they found that the *shank3^–/–^* embryos had significantly reduced expression of the RFP reporter from 1 to 3 dpf compared to *shank3*^+/+^ controls. The most prominent difference was observed at 1 dpf (Liu C.X. et al., 2018). Summary: SHANK3 is involved in dendritic spine development leading to impaired motor coordination, repetitive behaviors and altered social interactions. Shank3a/b knockouts in zebrafish have similar behavioral phenotypes but also have intestinal motility issues, linking the gut to CNS function. Combined with a transgenic line, these mutant zebrafish also had reduced differentiated neurons.

### SYNGAP1


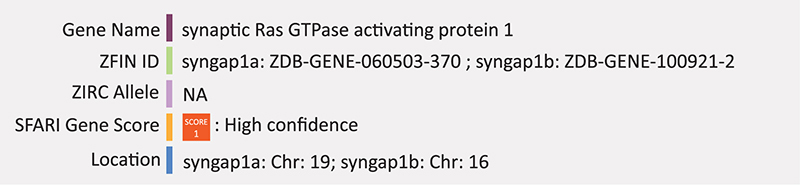


*SYNGAP1* encodes a synaptic Ras-GTPase-activating protein, involved in mediating the NMDA receptor activated RAS-signaling cascade and is highly expressed in prenatal brain regions (Tammimies, 2019). SYNGAP1 function is important in regulating post-synaptic density of dendritic spines where *SYNGAP1* haploinsufficiency causes pre-mature maturation of dendritic spines, a feature commonly associated with ASD (Gamache et al., 2020). Mutations in *SYNGAP1* are implicated in intellectual disability, ASD and epilepsy and approximately 50% of patients with loss-of-function mutations in *SYNGAP1* are diagnosed with ASD where tactile sensory deficits are a common feature of *SYNGAP1*-related ASD (Gamache et al., 2020).

Mice with a heterozygous *Syngap1* deletion spend more time in isolation and show complete lack of short-term social memory (Möhrle et al., 2020). Further, *Syngap1* mutant mice and rats show altered exploratory activity and behavioral inflexibility (measured by spontaneous alterations in a T-maze), and impaired whisker-evoked tactile sensory processing (Möhrle et al., 2020). Heterozygous conditional-knockout of *Syngap1* in the mouse hippocampus results in premature development of dendritic spine synapses, which is thought to be a major feature of ASD etiology (Clement et al., 2012).

Zebrafish have two *SYNGAP1* orthologs, *syngap1a* and *syngap1b*, each with at least two protein coding transcripts. A study using morpholino knockdown models of *shank3a* and *syngap1b* in parallel shows similar phenotypes with respect to ASD traits. Both knockdown models have increased cell death throughout the embryonic nervous system including the midbrain, hindbrain and spinal cord, altered brain morphology and seizure like behaviors (Kozol et al., 2015). Excitatory and inhibitory neurotransmission was compared between knockdowns using transgenic embryos marking excitatory neurotransmitter expression (Tg [vglut2:dsRed]) and counter stained with primary antibodies targeting GABA to mark inhibitory neurotransmitter expression. Significant decreases in GABAergic neurons were seen in the midbrain and hindbrain of morphant embryos, while excitatory neurons were only significantly decreased in the hindbrain. Transgenic embryos marking caudal primary neurons, motor neurons and Rohon beard sensory neurons (Tg [SaigFF213A]) were also used but both morphants and controls showed stage-appropriate expression (Kozol et al., 2015). Summary: SYNGAP1 is involved in NMDA-mediated Ras signaling at the synapse in the early developing brain, coordinating post-synaptic densities and dendrite maturity. In zebrafish syngap1a/b knockdown has increased cell death and a significant decrease in GABAergic neurons.

## Other Genetic Models of ASD

Zebrafish have been used in combination with other ASD-risk genes to study the molecular basis of autism and there have been a number of studies characterizing the function and expression patterns of other autism susceptibility gene orthologs in zebrafish. For example, the function of one of the original ASD susceptibility genes, aptly named *autism susceptibility candidate 2* (AUTS2), was first characterized in zebrafish using morpholino knockdown (Oksenberg et al., 2013). Expression of *auts2* in zebrafish is expressed throughout the brain during development and shows similar expression patterns to what has been previously characterized in mice. *Auts2* morphants display a developmental phenotype characterized by smaller head and body and reduced movement. *Auts2* morpholino injection in transgenic Tg[HuC:GFP] embryos revealed a reduction in sensory and motor neurons of the spinal cord and developing neurons of the midbrain and cerebellum suggest the potential regulatory role of *auts2* in neurodevelopment (Oksenberg et al., 2013).

Significant decreases in MET protein levels are often found in the cerebral cortex of ASD patients suggesting that disrupted Met signaling may contribute to the deficits in cerebellar growth and cell motility associated with autism (Campbell et al., 2007). Morpholino knockdown of zebrafish ortholog *met*, reveals its function in specification of ventricular zone progenitor cells. Furthermore, Tg[HuC-GFP] transgenic embryos marking newly differentiated neurons revealed *met* morphants have reduced proliferation in the developing cerebellum. Transgenic Islet1-GFP embryos (Tg[islet1:GFP]), which labels migrating facial motor neurons (FMN), show reduced hindbrain cell migration, overall supporting the role of *met* in cerebellar development and FMN migration (Elsen et al., 2009).

Experiments in zebrafish indicate that KCTD13 could be a major driver of the microcephalic and macrocephalic phenotypes associated with 16p11.2 CNVs observed in ASD. Morpholino knockdown of zebrafish *kctd13* results in increased neural progenitor cell number and increased brain size, whereas overexpression of human KCTD13 transcripts is associated with microcephaly (Golzio et al., 2012). Still, this connection requires further investigation, as the macrocephalic phenotype was not recapitulated in *kctd13* mutants of a later study (Escamilla et al., 2017).

Expression of *rbfox1l* was previously characterized in embryos and found to be expressed exclusively in cardiac and skeletal muscle (Gallagher et al., 2011). Contrastingly, *rbfox1l* expression has recently been characterized in the adult zebrafish brain using immunohistochemistry with anti-Rbfox1l antibodies. Here expression was restricted to populations of dorsal telencephalic neurons and the Purkinje cell layer of the cerebellum (Ma et al., 2019).

Wild-type and *reelin*^Δ^ ^28–/–^ mutants have similar measurements for anxiety indicating anxiety behavior is not affected by *reelin* mutation. However *reelin*^Δ^ ^28–/–^ mutants do display a selective reduction in preference for social novelty (Vecchia et al., 2019). This was paralleled by an increase in serotonin receptor signaling in the hindbrain. However, applying buspirone, a 5HT1A agonist that decreases 5-HT concentration at the synapse, did not rescue the mutant phenotype and therefore increases in serotonin signaling do not necessarily underpin the social preference phenotype (Vecchia et al., 2019).

It should be noted that some of the above genes, while interesting studies have been conducted on their impact on brain development with respect to ASD, have lower SFARI gene scores. Namely, *kctd13*, with a SFARI gene score of 3 (suggestive evidence) and *met* and *rbfox1*, which have gene scores of 2 (strong candidate).

## Non-Genetic Zebrafish Models of ASD

### Valproic Acid

Valproic acid (VPA) is a drug that is known to induce autism like effects in animal models. It is typically used as an anticonvulsant administered to individuals who experience seizures, however embryonic exposure to valproic acid has been linked to ASD. A population-based study of children born in Denmark between 1996 and 2006 found that the risk of ASD in children who were exposed to VPA *in utero* was 4.42% compared to the absolute risk of 0.48% in all children (Christensen et al., 2013). Other independent cohort studies have also found an increased risk of Autism in children prenatally exposed to VPA (Bromley et al., 2013; Wood, 2014). Animal models have linked embryonic VPA exposure to ASD-like behaviors as well as neurodevelopment defects. For example, maternal exposure of VPA has been shown to lead to autism-like behaviors and a reduction in mature neurons of the pre-frontal cortex and cerebellum of non-human primates (Zhao et al., 2019) and VPA exposure negatively affects the serotonergic system in rats as early as the progenitor cell stage (Dufour-Rainfray et al., 2010). Interestingly, the valproic acid rat model of autism also mimics the microbiome features of autism. Rats administered VPA during pregnancy have altered gut microbial profiles, fecal metabolite potential and reduced diversity of fecal microbes (Liu F. et al., 2018). It should be noted however, that the microbiome of the offspring of these rats was largely preserved. Valproic acid has been linked to ASD in other animal models including mice and prairie voles (Ornoy et al., 2019; Sailer et al., 2019).

Recently, zebrafish have been used as a model to study the effects of valproic acid on neurodevelopment. Valproic acid is a powerful histone deacetylase (HDAC) inhibitor and exposure to larval zebrafish results in phenotypic changes such as decreased neural progenitor cell proliferation in the telencephalon (Lee et al., 2013), reduced number of histaminergic neurons and decreased locomotion (Baronio et al., 2018), and failure of differentiation of serotonergic neurons (Jacob et al., 2014). Furthermore, Lee et al. (2018), performed an RNA-seq analysis on larval zebrafish after exposure to VPA and found transcriptional changes in ASD associated genes like *adsl, mdbs, tsclb*, and *shank3* as well as differential expression in 24 other candidate autism risk genes highlighting the use of valproic acid as a model to further investigate other mechanisms of autism. A similar approach was used by Liu et al., using valproic acid to study changes in shank3 transcripts through development (Liu et al., 2016).

### Germ-Free Models

Recent data demonstrates a strong correlation between gut microbiota and the etiology of ASD, with bacterially-derived metabolites from the gut linked to alterations in neurodevelopment and neural specific mRNA processing of the host (Irimia et al., 2014; Stilling et al., 2018; Sharon et al., 2019). Individuals with ASD are known to have gastrointestinal problems and distinct microbiome taxonomic profiles compared to neurotypical individuals (Sgritta et al., 2019). Studies in germfree mice demonstrate that microbiota are required for proper brain development (Borre et al., 2014; Sharon et al., 2019). Moreover, re-colonizing the gut of these germ-free mice with microbiomes of control mice ameliorates the associated deficits. However, mechanisms by which microbiota regulate nervous system development remain poorly defined. Presently there are many difficulties investigating the role of microbiota on the host that arise due to the complexity in microbiome taxonomy and variation between individual host microbiomes. Further complication arises in determining what stage is significantly impacted in neurodevelopment. Yet, due to the availability of access to early developmental stages, the zebrafish embryo presents a potential solution to at least part of this problem.

Recent evidence implicating autism with the gut microbiome brings attention to a new potential model. Gnotobiology is the study of model organisms in a germ-free environment or in the presence of known microorganisms. Gnotobiotic animals have been used to study the relationship between host and respective microorganisms for over 100 years (Nuttall and Theirfelder, 1897). Historically, the bulk of this research has been done using mice, however zebrafish present a relatively new and interesting opportunity for the study of the microbiome and its potential link to autism. Zebrafish are fertilized externally, develop rapidly and remain optically transparent during larval stages making them easy to derive germ-free and easy to analyze at very early stages of development. Because autism is a neurodevelopmental disorder, *in utero* development is a critical time period and access to these early stages in an animal model is an asset. Studies using germ-free zebrafish have described the role of the microbiota in the development of the immune system, digestive system and potential aspects of the enteric nervous system (Bates et al., 2006; Galindo-Villegas et al., 2012; Oehlers et al., 2012; Kanther et al., 2014; Marjoram et al., 2015; Rolig et al., 2018). A general overview of the current germ-free zebrafish phenotype is outlined in Table 1. Rawls et al. (2004) originally characterized the zebrafish gut microbiota and established a gnotobiotic zebrafish model up to late juvenile stages (6 dpf). They also described the host genetic and chemical responses to microbiota, many of which were conserved between fish and mice, and later established that the zebrafish and mouse microbiota can be reciprocally transplanted (Rawls et al., 2004, 2006). Recent studies have shown that the microbiota is implicated in ASD however, the use of germ-free zebrafish as a model to study autism is still in its infancy and there is still a general lack of understanding of the zebrafish microbiome and immune system in its relation to mice and humans. Studies thus far have been restricted to behavioral phenotypes, where caution should still be taken in drawing conclusions. Two recent studies show germ-free zebrafish display anxiety-like behavior and imbalanced hyper-locomotor activity with respect to time spent active compared to their conventionally raised, or microbially re-colonized counterparts (Davis et al., 2016; Phelps et al., 2017). Bruckner et al. (2020) recently showed that the gut microbiota is required for normal social behavior in zebrafish as well as to modulate neuronal features such as microglial morphology in early development.

**TABLE 1:** The germ-free phenotype in zebrafish.

Feature	Phenotype	References
Stress response	Lack of response to osmotic stress test with respect to cortisol levels	Davis et al., 2016
Innate immune system	Decreased neutrophil recruitment	Kanther et al., 2014
	Attenuated levels of intestinal pro-inflammatory mRNA	Marjoram et al., 2015
	Attenuated inflammation after intestinal injury	Oehlers et al., 2012
	Increased sensitivity to viral infection	Galindo-Villegas et al., 2012
Pancreas	Static number of pancreatic beta cells during development Increased glucose levels	Hill et al., 2016
Gut differentiation	Immature expression of glycoconjugates Lower levels of goblet cells Reduced level of protein macromolecular uptake	Bates et al., 2006
Behavior	Hyper-locomotor activity at 6 dpf	Davis et al., 2016
	No differences seen in hyper-activity at 1–6 dpf but increase at 10 dpf in dark period	Phelps et al., 2017

Even though these germ-free studies are restricted to the behavioral phenotype in relation to autism, it is interesting to note that often the resulting phenotype of the genetic knock out or pharmacological models of autism, such as those in mice, are comparable to the germ-free zebrafish phenotype (Table 1). It should be noted that it is not yet understood whether the link between ASD and the microbiome is due to a general lack of microbiota or the overgrowth of certain microbes and further research is required in order to resolve this. Nevertheless, the use of germ-free or specific-pathogen-free zebrafish could represent a future asset in ASD research, especially with respect to the idiopathic instances of ASD.

## Discussion

**FIGURE 2:**
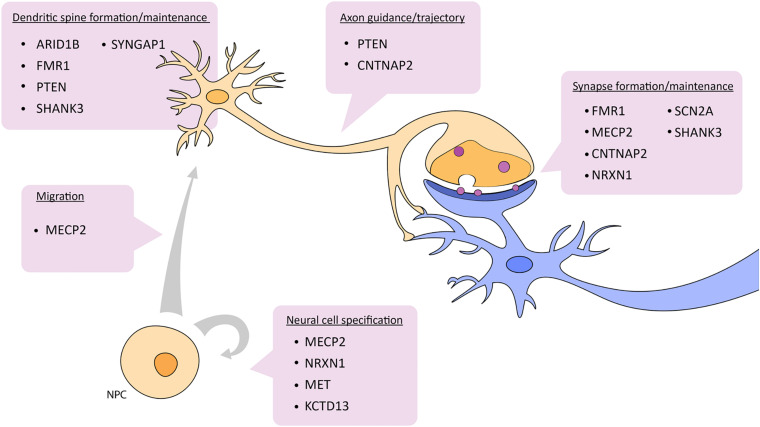
Depiction of which aspects of neuronal development and function are affected by ASD candidate genes described in this review. See text for details. NPC, neural progenitor cell.

Zebrafish have become a widely accepted animal model for translational neuroscience as they are primed for the study of complex brain disorders such as ASD. Beyond being genetically tractable and sharing significant genetic and physiological homology with mammals, zebrafish present similar behavioral phenotypes to mice and humans after manipulation of candidate autism risk genes. There are also several genes not actively being pursued yet have a clear link to ASD. It would be useful to study different ASD risk genes in combination in order to understand how they potentially interact. For example, many of the candidate genes mentioned above are known targets of CHD8, a candidate ASD risk gene and also part of the WNT signaling pathway. In a broader example, genes like NRXN, SHANK3, and PTEN interact with each other in the cellular pathway controlling synaptic function (Quesnel-Vallières et al., 2019) (Figure 2). Further, these two cellular pathways, WNT signaling and synaptic function, are highly integrated. Studying the pathways of which ASD risk genes belong opens up avenues to other genes in these pathways and may aid in the overall understanding of the genetic and molecular basis of ASD.

The alternative splicing of microexons is another school of thought behind the molecular basis of ASD. Dysregulated or aberrant splicing of microexons have been seen in the brains of autistic patients as well as in mouse models and these are often accompanied by expression changes in splicing factors such as SRRM4 and or RNA-binding proteins like RBFOX1 and MECP2 (Irimia et al., 2014; Quesnel-Vallières et al., 2016, 2019). These studies have yet to be replicated or expanded on in zebrafish but considering their semi high-throughput nature and general tolerance for genetic manipulation zebrafish would be highly amenable to this type of study.

Finally, the utility of zebrafish in behavioral neuroscience is increasing as the development of behavioral assays and collection of behavioral data is improving. Tools like DanioVision, an observation chamber designed specifically for the observation of zebrafish larvae, make the collection of behavioral data more replicable between experiments and even labs. The DanioVision system is developed by Noldus IT who also produce the DanioScope, a scope designed to measure zebrafish embryo activity and EthoVision XT, for video tracking of adult fish. Other commercially available software packages include ZebraLab (Viewpoint) designed for high-throughput screening of embryos, or Stytra, an open source software package (Štih et al., 2019) designed for tracking behavioral zebrafish experiments. Using various tools like this allows zebrafish behavioral phenotypes to be highly quantifiable.

Overall, zebrafish present an increasingly effective animal model for translational neuroscience and ASD research with many unexplored avenues of study. For example, knockout of *Pten* in mice results in increased size and abundance of axonal projections, number of dendritic spines and number of presynaptic vesicles. The work in zebrafish brain is limited to size phenotype. In this case, crossing the zebrafish *Pten* mutant line to a transgenic line that reports on dendritic cells or the synapse could provide further verification or insight; much like the *Cntnap2a/b* mutant zebrafish line whose GABAergic neurons fail to migrate dorsally. There are transgenic reporter lines including olig2:eGFP, which fluorescently marks the migration of oligodendrocyte precursor cells, or Tg[HuC:GFP], which fluorescently marks neurons through differentiation, that are available in zebrafish; both of which, are relevant to the molecular biology of ASD (Boyd et al., 2015; Castora, 2019). As the potential for reproduceable behavioral experiments increases, and the number of genetic models of ASD grows, zebrafish should be expected to make valuable contributions to our understanding of the genetic and environmental basis of ASD.

## Author Contributions

VR wrote the manuscript. TVR edited the manuscript. All authors created the concept.

## Conflict of Interest

The authors declare that the research was conducted in the absence of any commercial or financial relationships that could be construed as a potential conflict of interest.
