# Corrigendum: Using Zebrafish to Model Autism Spectrum Disorder: A Comparison of ASD Risk Genes Between Zebrafish and Their Mammalian Counterparts

**DOI:** 10.3389/fnmol.2021.695317

**Published:** 2021-05-13

**Authors:** Victoria Rea, Terence J. Van Raay

**Affiliations:** Dept of Molecular and Cellular Biology, University of Guelph, Guelph, ON, Canada

**Keywords:** ASD, autism, genes, behavior, zebrafish, human, mice

In the original article, Mueller et al. (2011) and Mueller (2012) were not cited in the article. The citation has now been inserted in Figure 1 legend, and should read:

Figure 1. Comparison of homologous regions of the **(A)** zebrafish **(B)** mouse and **(C)** human brains. Am: amygdala; Ce: cerebellum; Ctx: cortex; Dp: dorsal pallium; Hip: hippocampus; Lp: lateral pallium; Mp: medial pallium; Th: thalmus; Vp: ventral pallium. Zebrafish image in **(A)** adapted from Mueller et al. (2011) and Mueller (2012).

The authors apologize for this error and state that this does not change the scientific conclusions of the article in any way. The original article has been updated.

## References

[B1] Mueller T. (2012). What is the thalamus in zebrafish? Front. Neurosci. 6:64. 10.3389/fnins.2012.00064 22586363 PMC3345571

[B2] Mueller T. Dong Z. Berberoglu M. A. Guo S. (2011). The dorsal pallium in zebrafish, *Danio rerio* (Cryprinidae, Teleostei). Brain Res. 1381, 95–105. 10.1016/j.brainres.2010.12.089 21219890 PMC3052766

